# CloudAligner: A fast and full-featured MapReduce based tool for sequence mapping

**DOI:** 10.1186/1756-0500-4-171

**Published:** 2011-06-06

**Authors:** Tung Nguyen, Weisong Shi, Douglas Ruden

**Affiliations:** 1 Computer Science Department, Wayne State University, US; 2 Institute of Environmental Health Sciences, Wayne State University, US

## Abstract

**Background:**

Research in genetics has developed rapidly recently due to the aid of next generation sequencing (NGS). However, massively-parallel NGS produces enormous amounts of data, which leads to storage, compatibility, scalability, and performance issues. The Cloud Computing and MapReduce framework, which utilizes hundreds or thousands of shared computers to map sequencing reads quickly and efficiently to reference genome sequences, appears to be a very promising solution for these issues. Consequently, it has been adopted by many organizations recently, and the initial results are very promising. However, since these are only initial steps toward this trend, the developed software does not provide adequate primary functions like bisulfite, pair-end mapping, etc., in on-site software such as RMAP or BS Seeker. In addition, existing MapReduce-based applications were not designed to process the long reads produced by the most recent second-generation and third-generation NGS instruments and, therefore, are inefficient. Last, it is difficult for a majority of biologists untrained in programming skills to use these tools because most were developed on Linux with a command line interface.

**Results:**

To urge the trend of using Cloud technologies in genomics and prepare for advances in second- and third-generation DNA sequencing, we have built a Hadoop MapReduce-based application, CloudAligner, which achieves higher performance, covers most primary features, is more accurate, and has a user-friendly interface. It was also designed to be able to deal with long sequences. The performance gain of CloudAligner over Cloud-based counterparts (35 to 80%) mainly comes from the omission of the reduce phase. In comparison to local-based approaches, the performance gain of CloudAligner is from the partition and parallel processing of the huge reference genome as well as the reads. The source code of CloudAligner is available at http://cloudaligner.sourceforge.net/ and its web version is at http://mine.cs.wayne.edu:8080/CloudAligner/.

**Conclusions:**

Our results show that CloudAligner is faster than CloudBurst, provides more accurate results than RMAP, and supports various input as well as output formats. In addition, with the web-based interface, it is easier to use than its counterparts.

## Background

The rapid development of new sequencing technologies helps improve the accuracy as well as scope of many biological applications such as the assembly of genomes, transcriptomes (RNAs), or ChIP-Seq (chromatin-immunoprecipitation followed by next-generation DNA sequencing). Most of these applications execute the read alignment as their first step. Therefore, the sequence alignment is the most important and fundamental part to almost all applications of sequencing analysis.

New sequencing technologies in genomics create incredible amounts of data to process at a lower cost per nucleotide. Manufacturers are constantly increasing output in terms of the number of reads, increasing read length, as well as working to improve read quality. While it took 10 years and over $3 billion dollars to produce a first draft of the human reference genome (approx. 3.5 billion base pairs), the current generation of sequencing instruments is able to generate hundreds of billions of bases in only a few days. It is projected that this output will continue to increase dramatically over the next few years at a rate much faster than Moore's Law, a doubling every year, which is the approximate rate of increase in the semiconductor field over the past 40 or so years. For example, the latest sequencer from Illumina, the HiSeq 2000, is able to generate 25 Gb(gigabases)of sequence per day. In terms of price, in comparison to the prior model, the GA sequencer, the cost per base on the HiSeq is actually substantially reduced by as much as 8 times [1]. However, this is still the second-generation sequencing. The third-generation single molecule sequencing instruments are beginning to be introduced by Pacific Biosciences at a much reduced reagent cost and longer sequences.

These extensive genetic informational datasets create many serious problems and challenges for the popular alignment tools such as bowtie [2], RMAP [3,4], MAQ [5], bwa [6,7], etc. The first challenge is performance. As the data grows it is taking an increasing amount of time to compile, search and analyze and radical new approaches are required that would ensure project scalability. The second issue is the enormous capital expense for equipment that typically has 6 months as its state of the art half-life. Both computing and sequencing technologies advance at a very fast pace. To keep up with this pace, bio-organizations have to spend much money on replacing or updating devices.

In computer science, Cloud Computing has recently emerged as an evolutionary model to accommodate storage and computing service as a utility. Cloud providers offer different computing services to users through the Internet. Cloud users only pay for the resources (computing, bandwidth, etc) they actually consume without worrying about the maintenance expense, provisioning resources for future needs, taking care of availability, and reliability issues. The price is based on the time and types of services. As a result using Cloud Computing services is a recent and very promising solution in bioinformatics to deal with the issues related to storage and computation [8]. With Cloud solution, biologists don't need to equip and maintain powerful and high capacity servers for their analysis as before. They can securely store their data in the Cloud with high availability, and can have thousands of on-demand powerful computers ready to run their analysis. Nevertheless, to use Cloud, users need to be trained a little bit, and they are also required to have a stable high-speed Internet connection to the service providers.

The Cloud Computing solution, however, just enables the flexible and scalable infrastructure to deal with storage and computational issues. To deal with performance and scalability when processing a huge amount of data, we need to have a special parallel programming model. Recently, Google has designed a parallel computing framework called Mapreduce [9] which can scale efficiently many thousands of commodity machines. These commodity machines forming a cluster can be accessed by users in an institution or can be rented over the Internet through utility computing services. Actually, the idea of this framework is not new since it has already been used in traditional functional programming languages such as Haskell, Lisp, Erlang, etc.

**Figure 1:**
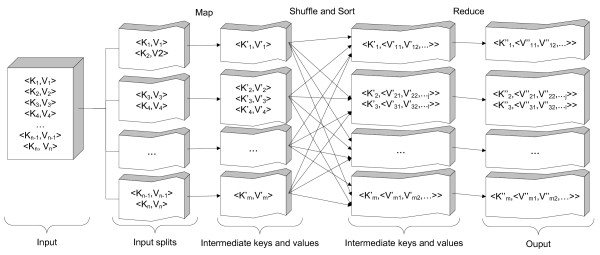
**The MapReduce framework**.

The basic idea of the MapReduce framework is shown in Figure 1. The data that need to be processed is divided into "input splits". Each split contains many records in a key-value pair structure <*K*,*V *>. The *map *blocks (a piece of code defined by software developers based on the application business) map these input key-value pairs into other intermediate key-value pairs. This intermediate data is then sorted and grouped together based on the keys. As a result, the input of the reduce blocks is a key with a collection of values. The *reduce *blocks (also developed by MapReduce programmers) then produce the final results in the form of key-value pairs as well. One very important feature enabling MapReduce to process a huge amount of data efficiently is that all *maps *and *reduce *blocks are executed concurrently. There are two main phases though: *map *and *reduce*. As we can see from the figure, all *map *tasks need to finish before running any *reduce *tasks.

There are many different implementations of the MapReduce framework such as Hadoop, Phoenix, Disco, Mars, etc. In developing our tool, CloudAligner, we chose Hadoop http://hadoop.apache.org since it is open-source (easy to fine tune), written in Java (high portability) and widely used in both academy and industry.

There have been some initiatives towards this trend of using Hadoop such as CloudBurst [10], SeqMapreduce [11], Crossbow [12], etc. The results are very promising. These tools can provide better performance and web-based interface which is easier to use than the command line interface of many existing tools.

However, in spite of these promising features, these Cloud-based applications do not significantly improve its functionality. Nor do they offer a variety of user-friendly features or interfaces needed to popularize them. For instance, the common functions that are often implemented in well-established on-premises alignment tools are bisulfite sequencing and pair-end mapping. These techniques are used for detecting genome variations such as single nucleotide polymorphisms (SNP) and large-scale structural variations, which are very important in biological analyses. The CloudBurst, for example, doesn't support either of these features. It also doesn't support the fastq input format which is a very common output of current sequencers. In addition, its interface is a command line style which is not very user-friendly. Another MapReduce-based software, SeqMapReduce, is a performance improvement version of CloudBurst, but its website and code are in-accessible. Crossbow is the read mapping and SNP calling software that runs in the Amazon EC2 cloud. It consists of a set of Perl and shell scripts that allow Bowtie and SOAPsnp to run on Cloud. Crossbow has a very nice and friendly web interface created with the aid of JotForm, a web-interface creation tool. However, since its biological functionalities depend entirely on other tools (Bowtie and SOAP-snp), it inherits their shortcomings too. For example, Bowtie can only allow at most 3 mismatches in its mapping and was only designed for short reads. Therefore it can't improve or fine tune the core functional algorithms.

Consequently, we developed CloudAligner to address such limitations of the existing tools and also to advocate a Cloud and MapReduce-based solution for genomic problems. Especially, CloudAligner is designed to achieve better performance, longer reads, and extremely high scalability. It has more common functions such as bisulfite (BS) and pair-end mapping as well as a friendly user interface, and it supports more input as well as output formats.

## Software Design

**Figure 2:**
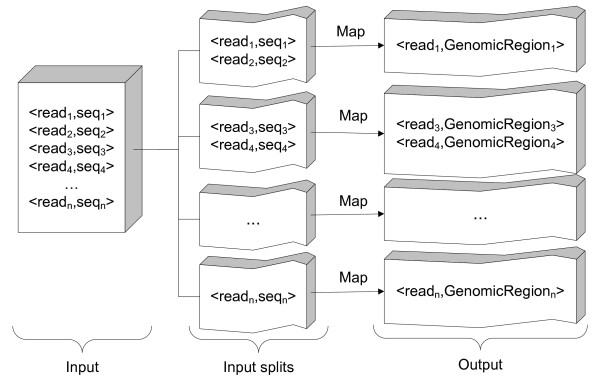
**CloudAligner architecture**.

Figure 2 shows the overall architecture of our tool. Not following the traditional MapReduce model like most other tools, CloudAligner does not have the reduce phase. The mapping algorithm (the popular seed-and-extend alignment algorithm) is implemented entirely in map tasks. By doing this, we don't have to spend time on operations such as shuffling and sorting of intermediated data. Also, parts of the final results can be obtained with this method as long as at least one map task successfully finishes since each map task aligns a small set of read on the whole genome. It is completely independent from other map results. This property is very beneficial especially for time-consuming jobs and the "pay-as-you-go" model of the Cloud because when the job fails, we can still have part of the result and only need to re-execute and pay for mapping the failed parts again.

There are two main input files for CloudAligner as the other alignment tools: the reference file and the read file. The reference files are normally in the fasta format while the read files can be in the fasta or fastq format. Both are changed into the serialized files (to be easily processed over the network) and copied to the HDFS or Amazon S3.

When executing, CloudAligner cuts the read file into smaller chunks called input splits (each read contains many read sequences) and distributes them to the mappers. Each mapper aligns its input split onto the whole reference genome file.

In terms of functionality, Table 1 highlights the supported features of CloudAligner in comparison to those of RMAP and CloudBurst. As shown in the table, our software has all fundamental features that a full-featured sequence mapping tool should have.

**Table 1:** Compare CloudAligner features with its counterparts

	CloudAligner	CloudBurst	RMAP
Mismatch Mapping	✓	✓	✓

Bisulfite Mapping	✓		✓

Pair-end Mapping	✓		✓

Fastq input	✓		✓

SAM output	✓		

Executable in Cloud	✓	✓	

This table summarizes the features of CloudAligner and other closely related tools.

## Experimental Results

### Evaluation criteria

We are going to evaluate CloudAligner in term of performance and accuracy. The performance metric is actually measured as the execution time of the tools. The accuracy is the number of reads that are mapped uniquely on the reference genome. To measure these two metrics, we built a Hadoop cluster of 13 nodes as a testbed for our experiment. The configuration of machines in our testbed is shown in Table 2. In the following experiments, the time to convert data to the Hadoop format and the time to move them into Hadoop Distributed File System (HDFS) or Amazon S3 are excluded.

**Table 2:** The detail configuration of machines of the main testbed

Type	Machines #	CPU	Memory	HDD	OS
Server	1	4 cores AMD 2GHz	6GB	250GB	64 bits Ubuntu Server 9.04
Server	12	1 core Intel Xeon CPU 2.80GHz	4GB	40GB	64 bits CentOS

This table details the configuration of our main Hadoop testbed.

### Mapping performance

As CloudBurst is also a Hadoop MapReduce based alignment tool, and the CloudBurst's paper [10] has shown the performance improvement (in term of speedup) over RMAP, we would like to compare our performance with it only. It is noteworthy that in our map task, we adopted the seed-and-extend mapping algorithm with different patterns in the seeds like RMAP. However, CloudAligner was developed in Java and doesn't have the limitation of using 64bit machine as RMAP.

**Figure 3:**
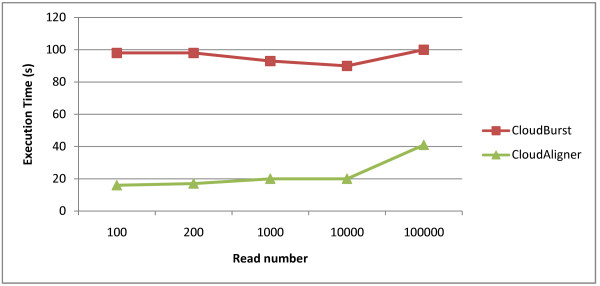
**The performance of CloudBurst and CloudAligner on small data**.

In this experiment, we ran both CloudAligner and CloudBurst on the same system with the same data set. The data set is obtained from the CloudBurst website. Figure 3 shows the performance results of both types of software with a different number of reads (the same reference file). The × axis in the figure is the number of reads, and the y axis is the execution time in second. From the figure, we can see that CloudAligner is 60 to 80% faster than CloudBurst.

**Figure 4:**
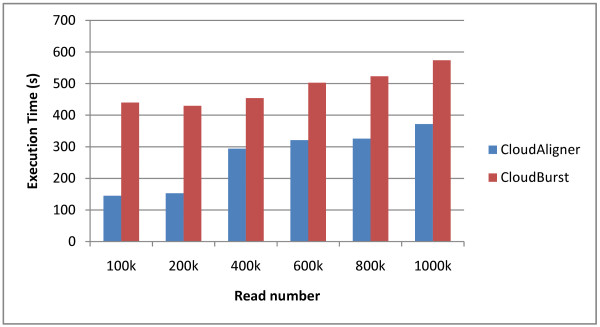
**The performance of CloudBurst and CloudAligner on larger data**.

We also did another experiment on the real data from the 1000 Genomes project. In particular, we mapped different subsets of the accession SRR035459 to the human chromosome 22 (50 Mbp) allowing up to 3 mismatches. The results of this mapping is shown in Figure 4. From the figure, we can see that the execution time of both CloudBurst and CloudAligner is proportional to the number of reads, and CloudAligner outperforms CloudBurst from 35 to 67%.

### Mapping accuracy

Since CloudAligner also employed the popular seed-and-extend algorithm like RMAP and Cloudburst, it generally inherits the limitations of this type of algorithm. Basically, this approach trades the accuracy for the performance. Instead of comparing the whole reads (with mismatches), the algorithms of this type only search for the shorted sequences called seeds. The accuracy of the result depends heavily on the seeds. The seed alignment can be consecutive or non-consecutive (template, pattern) matches.

To verify our results, we ran both CloudAligner and RMAP on the same set of data with equivalent seed information. With this type of experiment, we don't need to choose a very large workload because we only focus on the accuracy of the results. First, we ran CloudAligner with all the appropriate test reads (single-end, bisulfite, pair-end, fastq reads) of RMAP. Each data set has 100 reads with 25 bases in length. Our output files are the same as those of RMAP though with a different order. Second, we would like to test CloudAligner with another larger data set and longer reads to strengthen the soundness of our results. This time, RMAP and CloudAligner were executed (in mismatching mode) on the data includes 100,000 of single-end reads, and the reference genome is of the Streptococcus suis. Each read has 36 bases. CloudAligner only identified 74,208 unique maps while RMAP produced 74,291 unique maps. After carefully examining the extra 83 reads, we found that RMAP doesn't count the bad bases in the reads as mismatches which we should. Therefore it found more results with the same number of allowed mismatches.

### CloudAligner in Amazon EC2

**Figure 5:**
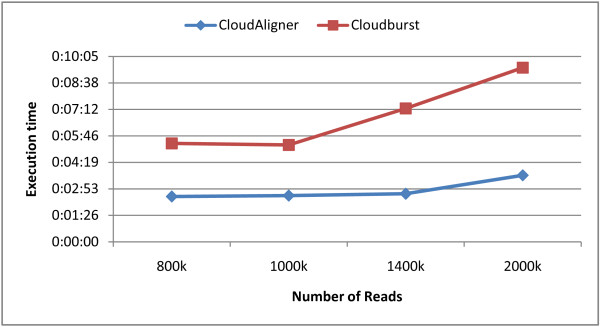
**The performance of CloudAligner and CloudBurst in Amazon Elastic MapReduce**.

To experience how our tool behaves in the real Cloud, we uploaded it to Amazon simple storage (S3) and created job flows in Amazon Elastic MapReduce to execute it. The execution time of CloudAligner and CloudBurst when processing different number of reads is expressed in Figure 5. It's safe to conclude that CloudAligner outperforms CloudBurst in the real cloud environment also.

**Figure 6:**
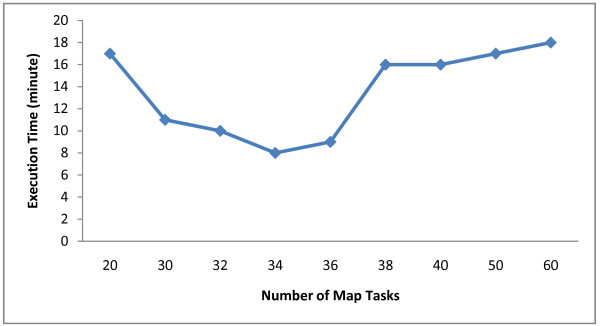
**The effect of the number of maps on the performance of CloudAligner**.

In addition to normal arguments such as read length, reference genome, input, output locations, CloudAligner (like other MapReduce applications) has the number of maps and reduces as its parameters. Therefore, we would like to study the effect of choosing different number of maps on the performance because, in our approach, there's no reduce task. Figure 6 shows the execution time of CloudAligner in the Amazon EC2 when mapping 2 millions of reads on the human chromosome 22 with different number of maps. The experiment was performed on 20 small EC2 instances. Thus we have totally 38 map slots (1 instance is used for the master node). The information in the figure suggests that the optimal number of input splits (maps) should be a little bit less than the maximum number of map slots. In this case, it should be either 34 or 36.

**Figure 7:**
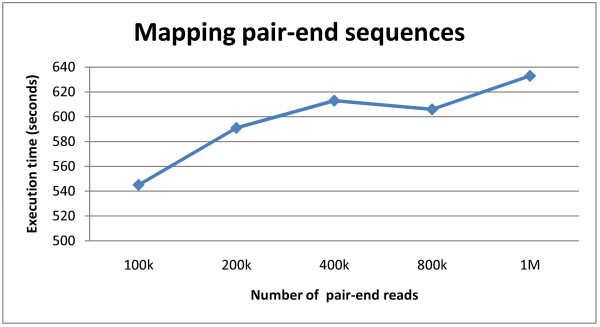
**Pair-end mapping in Amazon EC2**.

**Figure 8:**
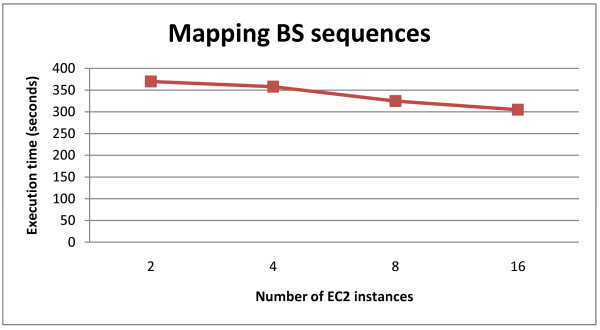
**Bisulfite mapping in Amazon EC2**.

The performance of pair-end and bisulfite mapping functions of CloudAligner is expressed in Figure 7 and 8 respectively. The pair-end read data is 76 bp in length and was obtained from the results of sequencing the African honey bee sample in our lab. Figure 7 shows the execution time when mapping different numbers of pair-end sequences (with quality scores) onto honey bee's chromosome 1 (A_mel 4.0). All of these mapping were taking place on 20 medium EC2 instances of Amazon Cloud. With the same number of instances, processing more read requires more time. For the BS mapping demonstration, we used the 100 k synthetic reads from BS Seeker. Figure 8 shows the execution time of this type of mapping with different number of EC2 small instances. Intuitively, the more instances we throw in, the faster the program is.

## Discussion

In general, in terms of performance, CloudAligner outperforms CloudBurst and RMAP. The performance gain over RMAP is mainly based on the scalability and parallel processing. With CloudBurst, the limitation of its approach is the network bandwidth. With CloudAligner, its limitation is in the computation power of the workers in Hadoop. Consequently, if we run CloudAligner on cluster of legacy machines with high speed network, we probably lose the performance advantage over CloudBurst. However, as shown in Table 2 the machines in our cluster are also not powerful at all. All of them only have a single core. Moreover, the interconnection between them is a brand new high-speed network (1 Gbps) since we put them in our newly built server room. Therefore, it is safe to conclude that in common cluster CloudAligner generally performs better.

We also developed the web-based interface for CloudAligner and hosted it at http://mine.cs.wayne.edu:8080/CloudAligner/.

From the website, users can upload the reads as well as the reference files in text format. The upload servlet automatically translates them into the Cloud format and uploads them to our Hadoop cluster. After having the files in the system, users can select them for the mapping together with common parameters such as the number of mismatch, seed, output format and so on. After finish mapping, the website creates a link to download the results.

CloudAligner can easily run on heterogeneous clusters. There are no restrictions on the hardware configuration of the machines constituting the cluster as long as they have enough memory to handle the small chunk of reads and reference genome assigned to them. To demonstrate this ability, we built a cluster of commodity machines as shown in Table 3 (except the master node) and ran CloudAligner on it to map 2 million reads on the human chromosome 2 (237 Mbp). It took 27 minutes and 18 seconds to finish this job. The only minor adjustment we need to do to handle larger data sets on outdated machines is to periodically inform the Hadoop system that our tasks are still alive. Otherwise, it assumes the nodes are dead and initiates the tasks on other nodes.

**Table 3:** The detail configuration of outdated machines for the heterogeneity tests

Machines #	CPU	Memory	HDD	OS
7	1 core Intel XEON CPU 1.80 GHz	512 MB	160 GB	32 bits CentOS
21	1 core Intel Pentium III	512 MB	20 GB	32 bits Ubuntu 8.04

This table details our local Hadoop testbed configuration for the heterogeneity experiment.

CloudAligner also offers the option to produce output files in both SAM [13] and BED6 formats to enable easier post processing analysis. For example, biologists can use the samtools [13] to identify SNP or INDEL in their samples or convert to the BAM file to have a visual view of the alignments.

Although CloudAligner theoretically has no limitations in read length as well as in number of mismatches, to efficiently deal with long reads, we should apply additional methods on the seeds such as the two-level techniques of Homer [14].

## Conclusions

With the improvement in sequencing technology, the data generated by the sequencers is becoming cheaper and better. Therefore, more data is increasingly being generated which leads to serious issues in storing and processing. Combining Cloud infrastructure and MapReduce framework together is emerging as one of the best solutions. However, the current tools of this trend are lacking the common features found in other popular tools making them unattractive to the users.

In this work, we built CloudAligner with the most common functions required for a mapping tool as well as an easy-to-use web-based interface to endorse the tendency of using Cloud and MapReduce. The summary of these functions is described in detail in Table 1. Moreover, we also designed and implemented a new approach to improve the performance of our tool. Our results indicate that significant improvement in the performance of alignment MapReduce-based tools can be achieved by omitting the reduce phase.

In the future, we plan to extend our tool to efficiently handle very long reads which will be generated by the next generation sequencers.

## Availability and Requirements

Project name: CloudAligner

Project home page and source code: http://cloudaligner.sourceforge.net/

The executable jar file: Additional file 1

Operating system(s): Linux

Programming language: Java 1.6.0

Other requirements: Any web browser

Licence: GNU GPL

## Competing interests

The authors declare that they have no competing interests.

## Authors' contributions

TN, WS and DR conceived the research and wrote the manuscript; TN developed the software and conducted the experiments. All authors has read and approved the final manuscript.

## Supplementary Material

Additional file 1 **CloudAligner.jar**. Executable java file that is used to run CloudAligner in both EC2 and local Hadoop system. Click here for file
